# A Genome-Wide Investigation of Copy Number Variation in Patients with Sporadic Brain Arteriovenous Malformation

**DOI:** 10.1371/journal.pone.0071434

**Published:** 2013-10-03

**Authors:** Nasrine Bendjilali, Helen Kim, Shantel Weinsheimer, Diana E. Guo, Pui-Yan Kwok, Jonathan G. Zaroff, Stephen Sidney, Michael T. Lawton, Charles E. McCulloch, Bobby P. C. Koeleman, Catharina J. M. Klijn, William L. Young, Ludmila Pawlikowska

**Affiliations:** 1 Center for Cerebrovascular Research, Department of Anesthesia and Perioperative Care, University of California San Francisco, San Francisco, California, United States of America; 2 Institute for Human Genetics, University of California San Francisco, San Francisco, California, United States of America; 3 Department of Epidemiology and Biostatistics, University of California San Francisco, San Francisco, California, United States of America; 4 Cardiovascular Research Institute, University of California San Francisco, San Francisco, California, United States of America; 5 Kaiser Northern California Division of Research, San Francisco, California, United States of America; 6 Department of Neurological Surgery, University of California San Francisco, San Francisco, California, United States of America; 7 Department of Medical Genetics, University Medical Center, Utrecht, The Netherlands; 8 Department of Neurology and Neurosurgery, Rudolf Magnus Institute of Neuroscience, University Medical Center, Utrecht, The Netherlands; 9 Department of Neurology, University of California San Francisco, San Francisco, California, United States of America; Medical College of Wisconsin, United States of America

## Abstract

**Background:**

Brain arteriovenous malformations (BAVM) are clusters of abnormal blood vessels, with shunting of blood from the arterial to venous circulation and a high risk of rupture and intracranial hemorrhage. Most BAVMs are sporadic, but also occur in patients with Hereditary Hemorrhagic Telangiectasia, a Mendelian disorder caused by mutations in genes in the transforming growth factor beta (TGFβ) signaling pathway.

**Methods:**

To investigate whether copy number variations (CNVs) contribute to risk of sporadic BAVM, we performed a genome-wide association study in 371 sporadic BAVM cases and 563 healthy controls, all Caucasian. Cases and controls were genotyped using the Affymetrix 6.0 array. CNVs were called using the PennCNV and Birdsuite algorithms and analyzed via segment-based and gene-based approaches. Common and rare CNVs were evaluated for association with BAVM.

**Results:**

A CNV region on 1p36.13, containing the neuroblastoma breakpoint family, member 1 gene (*NBPF1*), was significantly enriched with duplications in BAVM cases compared to controls (P = 2.2×10^−9^); *NBPF1* was also significantly associated with BAVM in gene-based analysis using both PennCNV and Birdsuite. We experimentally validated the 1p36.13 duplication; however, the association did not replicate in an independent cohort of 184 sporadic BAVM cases and 182 controls (OR = 0.81, P = 0.8). Rare CNV analysis did not identify genes significantly associated with BAVM.

**Conclusion:**

We did not identify common CNVs associated with sporadic BAVM that replicated in an independent cohort. Replication in larger cohorts is required to elucidate the possible role of common or rare CNVs in BAVM pathogenesis.

## Introduction

Brain arteriovenous malformations (BAVM) are a tangle of poorly formed blood vessels with abnormal connections between arteries and veins, with direct shunting of blood through a vascular nidus but without an intervening capillary bed. BAVMs are rare, occurring in less than 1% of the general population, but are a leading cause of hemorrhagic stroke in children and young adults. Although the majority of BAVMs arise sporadically, they also occur in patients with Hereditary Hemorrhagic Telangiectasia (HHT), a Mendelian disorder inherited in an autosomal dominant fashion and caused by mutations in one of three genes (*ACVRL1*, *ENG* and *SMAD4*) in the TGFβ signaling pathway [1], [2], [3], [4], [5].

Genetic risk factors have been implicated in susceptibility to non-HHT BAVM [6]. A linkage study in multiplex BAVM families of Japanese ancestry implicated three loci on chromosomes 5, 15 and 18 [7]. Inoue et al [8] performed linkage analysis in 6 BAVM affected pairs from 6 unrelated families, and reported suggestive linkage to 7 candidate regions (3q27, 4q34, 6q25, 7p21, 13q32–33, 16p12–13, and 20q11–13) with the strongest support for 6q25 (LOD = 1.88, P = 0.002). Candidate gene studies have suggested that common variants in *ACVLR1* [9], *IL1β* [10], *ITGB8* [11], and *ANGPTL4* [12] are associated with sporadic BAVM. Finally, several mouse models support the role of genetic mechanisms in BAVM development [13], [14].

Copy number variations (CNVs) represent a significant source of genetic variation. CNVs, defined as deletions or duplications of a segment of DNA sequence ≥1 kb in size compared to a reference genome, affect roughly 12% of the human genome [15]. *De novo* CNVs can be a potential genetic mechanism in sporadic diseases [15]. Recent studies have demonstrated association of rare and common CNVs with several diseases, including schizophrenia [16], [17], [18], [19], autism [20], [21], and amyotrophic lateral sclerosis [22], [23]. Mechanisms by which CNVs may influence gene function and thus disease susceptibility include gene dosage imbalances, altered messenger RNA (mRNA) expression levels or expression of truncated proteins with altered function [24].

Modern genome-wide arrays include probes for assessing CNVs, and CNVs can also be called using intensity signals from single nucleotide polymorphism (SNP) probes. However, accuracy of the current CNV calling algorithms varies considerably, yielding substantial false negative and false positive rates [25], [26]. A recent study evaluating the performance of five commonly used CNV calling algorithms concluded that PennCNV and Birdsuite are superior to others when considering overall reproducibility of calls and Mendelian consistency [27].

We hypothesized that CNVs (rare or common) may contribute to sporadic BAVM risk. To obtain reliable CNV calls for association analysis, we used two algorithms to call CNVs and focused on CNVs identified by both algorithms significantly associated with BAVM. Here we present the results of the first genome-wide association study (GWAS) of CNVs in patients with sporadic BAVM.

## Materials and Methods

### Ethics statement

All participants gave written informed consent, and the study was approved by the Committee on Human Research (CHR) at the University of California, San Francisco; Kaiser Permanente Northern California Institutional Review Board for the Protection of Human Subjects; and the University Medical Center Utrecht Medical Ethics Review Committee, The Netherlands.

### Sample recruitment

The initial cohort included sporadic BAVM patients (n = 371) recruited at the University of California, San Francisco (UCSF) or Kaiser Permanente Medical Care Plan of Northern California (KPNC) as part of a larger UCSF-KPNC Brain AVM registry. Of the 371 cases, 95 provided saliva and 276 cases provided blood specimens for DNA extraction. Controls included 216 healthy controls from a narcolepsy study [28] and 347 transplant donors from a kidney transplantation study [29]. All control participants provided blood specimens. Cases and controls were all of self-reported Caucasian race/ethnicity. The replication cohort comprised 184 Caucasian BAVM cases (37 cases from UCSF and 147 cases from the University Medical Center, Utrecht, The Netherlands) and 182 healthy Caucasian controls recruited for the Study Of Pharmacogenetics in Ethnically Diverse Populations (SOPHIE) [30]. AVM diagnosis, morphological, and clinical characteristics were recorded using standardized definitions [31], [32].

### Genotyping

The discovery cohort was genotyped using the Affymetrix Genome-Wide Human SNP array 6.0 (Affymetrix, Santa Clara, California), according to the manufacturer's protocols (http://www.affymetrix.com); both cases and controls were genotyped in the same laboratory at UCSF. The Affymetrix 6.0 array contains 906,600 SNP probes and 946,000 CNV probes. Of the CNV probes, 800,000 are evenly spaced along the genome and the remaining probes target 3,700 known CNVs [33].

### CNV calling and quality control filtering

#### Pre-CNV calling QC

We discarded samples with more than 5% missing genotypes, or which disagreed on computed and reported gender. For known or cryptic duplicates, the sample with the lower genotype call rate was dropped. Overall average genotyping call rate was 99%. A total of 338 cases and 510 controls passed pre-CNV calling QC filtering.

#### CNV calling

To identify deletions and duplications for the 22 autosomes, we used the version of the PennCNV algorithm optimized for CNV calls from the Affymetrix 6.0 array (http://www.openbioinformatics.org/penncnv/penncnv_tutorial_affy_gw6.html) and adjusting for genomic waves [34], [35]. At each marker, the B allele frequency (BAF), a measure of the normalized allelic intensity ratio, and log R ratio (LRR), a measure of the normalized total signal intensity are used together to infer copy number state. This algorithm combines these values, the distance between SNPs, and the population frequency of the B allele into a hidden Markov model (HMM) to identify autosomal deletions and duplications. For more precise modeling of the CNV events, PennCNV adopts a six-state definition [34]. To ensure reliability of the CNV calls produced by PennCNV, we also called CNVs using the Birdsuite algorithm. Birdsuite is a four-stage integrated analysis of SNPs and CNVs designed specifically for the Affymetrix 6.0 array. Birdsuite sequentially assigns copy number across regions of common copy number polymorphisms (CNPs) using Canary software, then calls SNP genotypes and identifies rare CNVs via HMM using Birdseye. Finally, copy number and SNP allele information are combined to provide an integrated genotype at every locus [36]. The Canary software determines copy number polymorphisms (CNPs) which are catalogued and present in more than 1% of 270 HapMap samples [33]. We used results from PennCNV as our primary findings and focused on top findings for which PennCNV and Birdsuite gave similar results as they are more likely to be genuine findings. Previous studies suggest that PennCNV is one of the optimal algorithms, and the use of more than one algorithm is highly recommended for CNV calling to reduce false positive calls [37], [38], [39].

#### Post-CNV calling QC

We retained only those CNVs that were called based on ≥20 markers in both PennCNV and Birdsuite (analysis with a ≥10 marker cutoff yielded similar results, data not shown). For PennCNV, to reduce the number of false positives, we removed outliers with respect to the LRR standard deviation (upper quartile+1.5×IQR), BAF-median greater than 0.55 or less than 0.45, BAF-drift >0.005 and waviness factor of greater than 0.04 or less than −0.04. We also removed samples that were outliers with respect to the number of CNVs per individual (>92 CNVs, based on upper quartile+1.5×IQR).

For CNVs called by Birdseye, we excluded CNVs with LOD <10 and samples with high sample-specific measures of noise (variance >2). CNPs assigned a copy number state equal to 2 (normal) by Canary and those that mapped to the sex chromosomes were removed. Only CNPs with high confidence score (<0.1) were included for analysis. The list of common CNPs generated by Canary was then merged with the list of CNVs generated by Birdseye into one master file. Samples with an excess number of CNVs called (>633/sample) were also removed from downstream analysis. Since CNVs may be artificially split by the CNV calling algorithm, for both PennCNV and Birdsuite, adjacent calls of the same type were combined into a single CNV if the gap between the calls was <20% of the total length of adjacent calls including the gap region. This resulted in a total of 26,355 CNVs across 270 cases and 457 controls for PennCNV and 27,657 CNVs across 289 cases and 443 controls for Birdsuite. CNVs overlapping telomeres, centromeres or segmental duplications were not removed but flagged as these regions are known to harbor spurious CNV calls. We also explored the results after excluding CNVs with >50% of their length overlapping segmental duplications.

### Statistical analysis

CNV size was compared between cases and controls using a two-tailed Mann-Whitney U test because of the non-normality of the data. We tested association of both rare and common CNVs with increased risk of BAVM. For tests of association of common CNVs, we used both a segment-based scoring approach and a gene-based approach [40], [41]. All coordinates are according to the human NCBI Build 36, hg18 reference sequence. All statistical analyses, except where otherwise noted, were performed using R version 2.10.1 software (www.rproject.org).

### Segment-based scoring approach

CNV regions (CNVRs) were defined using a segment-based scoring approach that scans the genome for consecutive markers to identify loci with significantly more CNVs in cases compared to controls. Each marker is tested for enrichment of CNVs in cases versus controls after correcting for multiple testing using a one-sided Fisher's exact test; this is done for duplications and deletions separately. We used principal component analysis (PCA) to model ancestry differences between cases and controls. PCA was performed by Eigenstrat v3.0 using SNP genotype calls from 72,456 unlinked markers distributed uniformly across the genome [42]. To confirm findings from PennCNV, CNVRs were defined in the same way using Birdsuite calls passing QC filtering. Only regions passing multiple testing correction using both PennCNV (adjusted for 91,083 tests for duplications and 80,663 tests for deletions) and Birdsuite (adjusted for 84,455 tests for duplications and 63,070 for deletions) were considered for downstream analysis. Because of the uncertainty in defining CNV boundaries when using intensity data from SNP arrays, CNVRs are defined by the union (total length encompassed by both algorithms). Association of CNVRs with BAVM was assessed by fitting a multivariate logistic regression model adjusting for age, sex and the top 3 principal components for population substructure. A CNVR was considered significantly associated with BAVM if P<10^−5^ in both PennCNV and Birdsuite. Finally, B allele frequency (BAF) and log R ratio (LRR) plots were manually examined for top BAVM-associated loci.

### Gene-based approach

We performed a gene-based analysis to assess for significant enrichment of CNVs overlapping known genes in BAVM cases compared to controls. This approach identifies CNVs that could be individually rare, or may disrupt different parts of specific genes that could be involved in important pathways and contribute to the etiology of BAVM. In addition, it allows combined analysis of rare and common CNVs impacting the same gene, thus allowing evaluation of CNV calls that might be missed by the segment-based approach.

To test for genes associated with BAVM, we examined CNVs overlapping genes plus 20 kb upstream and downstream of the gene boundaries. Significance was assessed using a one-sided Fisher's exact test correcting for the 1126 genes overlapping CNVs from both PennCNV and Birdsuite using the Bonferroni correction. Deletions and duplications were tested separately.

### Rare CNV analysis

To test the hypothesis that cases have a greater burden of rare large CNVs compared to controls, we performed a burden analysis, defining burden as either: 1) the total number of CNVs carried by an individual, or 2) the total number of genes spanned by those CNVs.

For rare CNV analysis, we only considered CNVs called using PennCNV and >100 kb. We removed individuals who were outliers with respect to the total number of CNVs called per individual and to the total kb span of the CNVs. Finally, common CNVs (present in >1% of the total sample) were excluded, as well as CNVs that overlapped by at least 50% of their length with previously described common CNVs (PLINK software version 1.06) [43]. The final dataset consisted of 732 rare large CNVs from 437 individuals (158 cases and 279 controls).

The CNVs were further stratified by type (deletions or duplications) and by size (100–200 kb, 200–500 kb, 500–1000 kb and >1000 kb). Permutation was used to test if the total number of CNVs carried by an individual as well as the total number of genes spanned by those CNVs was significantly higher in cases compared to controls (PLINK) [43].

Similar analysis was performed for rare CNVs restricting to each of seven candidate biological pathways relevant to BAVM based on prior human studies and animal models (TGFβ signaling including the 3 known HHT genes, Notch signaling, Vascular Endothelial Growth Factor (VEGF) signaling, Angiogenesis, Vascular Development, Inflammatory Response and Mitogen-Activated Protein Kinase (MAPK) signaling) [44], [45], [46], [47].

### Experimental validation and replication

To validate our top findings, we used several quantitative PCR (qPCR) assays. First, we used a commercially available probe (Applied Biosystems, Foster City, CA, Taqman Hs04206910_cn in *NBPF1*). qPCR was performed with 10 ng DNA in 10 µL reactions in triplicate with RNAseP internal reference control on an ABI7900HT thermocycler. For validation, we assayed 61 cases from the original cohort and evaluated concordance between copy numbers estimated by qPCR and by CNV calling algorithms (we did not have access to DNA from the original controls so we could not evaluate concordance in controls). For replication, we assayed 184 new BAVM cases (from Utrecht and UCSF) and 182 new controls. A negative control and at least 2 HapMap Caucasian reference samples were included on each plate (NA06991, NA06985 and NA12875). Threshold cycle (C_T_) values for the target and the reference generated by qPCR were imported into CopyCaller software (Applied Biosystems, Foster City, CA). Copy number of the target sequence was determined by comparing cycle threshold (C_T_) between locus probe and internal reference probe (ΔΔC_T_) using CopyCaller software (Applied Biosystems, Foster City, CA). We used the HapMap sample NA06991 as a calibrator sample.

Second, we designed three custom qPCR probes targeting the *NBPF1* gene (probe 1, 5′-CCGAAGCCCTAAATCTCAAC-3′ and 5′-ACGGCAAGGGACAATTGGCT-3′; probe 2, 5′-TTTGTGTCCGGAATGTGCCT-3′ and 5′- CCCTGCACTTACCCTTGTCC-3′; probe 3, 5′-TTTCTACCTGGCCCTGGTCT-3′ and 5′-CCCCAGCTACATTTCATGGCT-3′) and assayed them in 177 BAVM cases from Utrecht (123 cases overlapped the first replication cohort above). As above, a negative control and at least 2 Caucasian reference samples were included on each plate. Real time qPCR was performed with 20 ng DNA in 25 µL reactions in triplicate on an ABI7900HT thermocycler. CNVs were called using the ΔΔC_T_ method using the average ΔC_T_ of the total sample as the reference.

### Gene ontology and pathway analysis

The Database for Annotation, Visualization and Integrated Discovery (DAVID) v6.7b was used for analyzing functional classification, gene ontology (GO) and pathway analysis (http://david.abcc.ncifcrf.gov/) for BAVM-specific genes, defined as genes overlapping at least one CNV in cases and none in controls.

## Results

A total of 338 BAVM cases and 510 controls passed array QC. Cases were significantly younger than controls (mean age = 38.7 y±17.6 y and 50 y±14 y respectively, P<0.001). Gender distribution was similar between cases and controls (percentage of females: 54.4% cases, 49.8% controls, P = 0.19). Cases had an average BAVM size of 3 cm±1.6 (mean ± standard deviation); 16.85% had exclusively deep venous drainage and 38% presented with hemorrhage.

### CNV calling

Using PennCNV, we observed a total of 46,251 raw CNV calls across 338 BAVM cases and 510 controls, and 26,355 QC-filtered CNV calls across 270 cases and 457 controls. The average number of CNVs called per individual was significantly lower in cases compared to controls (34 vs. 37, P = 3.3×10^−9^). The overall median CNV size was significantly larger in cases compared to controls (40 kb in cases vs. 35 kb in controls, P = 1.2×10^−14^). For duplications, the average number of CNVs per individual did not differ between cases and controls. For deletions, the average number of CNVs called per individual was significantly smaller in cases compared to controls (Table S1); this was also the case for deletions using Birdsuite. However, for duplications using Birdsuite, we observed a higher average number of CNVs called per individual in cases compared to controls (Table S1). Since 26% of the cases provided saliva specimens for DNA extraction, we also compared the average number of CNVs called between cases with saliva specimens and cases with blood specimens. The average number of duplications called per individual was significantly higher in cases with blood specimens compared to cases with saliva specimens (16 vs. 12, P = 4.31×10^−6^). For deletions, the average number of CNVs called per individual did not differ between blood and saliva DNA.

### Segment-based analysis

**Figure 1:**
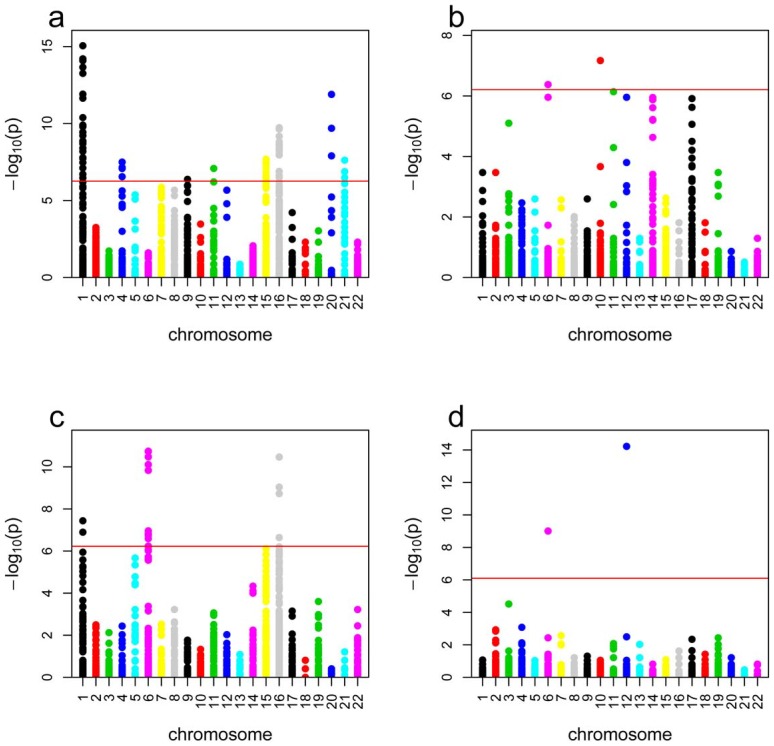
Plot of CNVR association with BAVM from the segment-based analysis. Chromosomes and -log p-values of CNVR association with BAVM are shown on the x and the y axes; respectively. The red horizontal line corresponds to the genome wide significance threshold corrected for multiple testing using the Bonferroni procedure. **a**. Duplications using PennCNV, **b**. Deletions using PennCNV, **c**. Duplications using Birdsuite. **d**. Deletions using Birdsuite.

Using PennCNV, we identified 11 CNVRs (9 duplications, Figure 1a, and 2 deletions, Figure 1b and Table 1) with significantly higher frequency in BAVM cases compared to controls (Fisher's Exact test after correcting for multiple testing, P≤1.02×10^−5^, Table 1). All 11 CNVRs overlapped at least one copy number locus with a frequency of >1% in the HapMap population [33]. Among those 11 CNVRs, only one deletion on chromosome 6 (Figure 1d) passed the correction for multiple testing using Birdsuite (P = 1.49×10^−9^).

**Table 1:** BAVM-associated CNVRs (PennCNV).

CNVR	Length, bp	Type	Cases	Controls	OR	P*	Overlapped genes
chr1: 16,741,950–16,987,299	245,349	Dup	64 (0.237)	26 (0.057)	5.14	2.70E-12	*AL035288,DQ585677, FLJ00313,KIAA0445, MST1,MSTP9, NBPF1,NBPF10*
chr1: 25,465,715–25,534,799	69,084	Dup	65 (0.241)	36 (0.079)	3.70	2.00E-09	*AX747205,RHD*
chr4: 2,281–52,126	49,845	Dup	35 (0.130)	13 (0.028)	5.08	2.05E-07	*ZNF595,ZNF718*
chr9: 68,101,177–68,126,440	25,263	Dup	17 (0.063)	1 (0.002)	30.53	4.19E-07	*AK311167,CR615453, CR626459*
chr11: 4,933,069–4,933,190	121	Dup	49 (0.181)	25 (0.055)	3.82	8.22E-08	*OR51A2*
chr15: 18,700,552–18,809,644	109,092	Dup	82 (0.304)	67 (0.147)	2.54	4.62E-07	*CR622584,DQ592463*
chr16: 32,756,399–33,638,076	881,677	Dup	62 (0.230)	49 (0.107)	2.48	1.02E-05	*BC038215,BC041879, DQ571479,DQ574674, IGH,IGHV,LOC440366, SLC6A10P,TP53TG3, TP53TG3b*
chr20: 1,508,252–1,508,999	747	Dup	34 (0.126)	6 (0.013)	10.8	2.03E-10	*SIRPB1*
chr21: 10,061,090–10,197,783	136,693	Dup	23 (0.085)	3 (0.007)	14.05	5.26E-08	*BAGE,BAGE1, BAGE2,BAGE5*
chr6: 103,841,338–103,841,522	184	Del	17 (0.063)	1 (0.002)	30.53	4.19E-07	*None*
chr10: 46,478,798–46,479,827	1,029	Del	56 (0.207)	32 (0.070)	3.47	6.77E-08	*ANXA8*

* P values are calculated using a one-sided Fisher's exact test.

Among the 9 duplications passing the correction for multiple testing using PennCNV, parts of 3 CNVRs on chromosomes 16, 15 and 1 were also identified by Birdsuite (P<2×10^−5^). CNVRs were then defined as the union (total length encompassed) of the CNVRs found by the two algorithms. We further assessed the association of the 4 CNVR with BAVM using a multivariate logistic model adjusting for age, sex and the top 3 principal components of population structure (Table 2). Only one CNVR on 1p36.13 was significantly associated with BAVM in the multivariate model using both PennCNV and Birdsuite.

**Table 2:** Multivariate regression model of association of three top CNVRs with BAVM.

				PennCNV				Birdsuite			
CNVR	Length	Type	No. probes	Cases	Controls	OR	P	Cases	Controls	OR	P
chr1: 16,741,950–17,055,024	313,074	Dup	123	71 (0.263)	34 (0.074)	4.1	2.20E-09	53 (0.18)	14 (0.03)	6.9	2.00E-09
chr15: 18,700,552–18,809,644	109,092	Dup	18	82 (0.304)	67 (0.147)	2.7	6.55E-07	47 (0.16)	25 (0.06)	3.3	1.37E-05
chr6: 103,841,338–103,841,522	184	Del	5	17 (0.063)	1 (0.002)	31.3	9.81E-04	31 (0.11)	4 (0.009)	12.9	3.91E-06
chr16: 32,756,399–33,638,076	881,678	Dup	146	62 (0.23)	49 (0.11)	2.6	1.8E-05	153 (0.53)	199 (0.45)	1.4	3.42E-02

This table summarizes the associated CNVRs with BAVM using a multivariate logistic regression model adjusting for age, sex, and the top three principal components.

After removing CNVs that overlapped segmental duplication regions, none of the CNVRs were significantly associated with BAVM in the multivariate model.

We also evaluated concordance between CNV calls made by PennCNV and Birdsuite for each CNVR. For the CNVR on 1p36.13, 38 of 105 subjects (36%) called duplications by PennCNV were also called duplications by Birdsuite. For the CNVR on 15q11.2, 66 of 149 samples (44%) called duplications by PennCNV were also called duplications by Birdsuite. For the CNVR on 16p11.2, 72 of 111 (65%) subjects called duplications by PennCNV were also called duplications by Birdsuite. For the CNVR on 6q16.3, only 1/18 samples (6%) were concordant for a deletion call.

### Gene-based analysis

To complement the segment-based analysis with a CNV analysis that is less sensitive to CNV definition, we performed a gene-based analysis, including the gene and 20 kb upstream and downstream. We first examined CNVs in the three HHT genes (*ACVRL1*, *ENG* and *SMAD4*). None of the CNVs called by PennCNV overlapped any of the HHT genes in BAVM cases.

We then performed a genome-wide analysis; a total of 1126 genes overlapped CNVs called by both PennCNV and Birdsuite. Thirty gene transcripts showed significant enrichment of CNVs in cases compared to controls using PennCNV after correction for multiple testing (Table 3). Only one gene, *OR4K1* (olfactory receptor, family 4, subfamily K, member 1) was significantly enriched for deletions in cases compared to controls (P = 1.13×10^−6^); however, this was not supported by Birdsuite analysis. Among duplications, the *NBPF1* gene on chromosome 1 was significantly associated with BAVM in both PennCNV and Birdsuite (Table 3 and Table S2); *NBPF1* is located within the BAVM-associated CNVR on chromosome 1 from the segment-based analysis. Figure S1 shows the duplication CNVs called by PennCNV at 1p36.13 in cases and controls [48]. 486 genes overlapped CNVs from both PennCNV and Birdsuite after excluding CNVs with >50% of their length overlapping segmental duplication regions; none of these genes were significantly associated with BAVM after correcting for multiple testing.

**Table 3:** Genes overlapping BAVM-associated CNVs (PennCNV).

Gene	Chr	Type	Cases	Controls	Proportion Cases	Proportion Controls	OR	P (unadjusted)*	P (Bonferroni_ adjusted)**
*NBPF10*	1	Dup	48	8	0.18	0.02	12.09	8.20E-15	9.23E-12
*NBPF1*	1	Dup	50	10	0.19	0.02	10.13	2.17E-14	2.44E-11
*RHD*	1	Dup	65	36	0.24	0.08	3.70	2.00E-09	2.25E-06
*C1orf63*	1	Dup	64	36	0.24	0.08	3.63	3.95E-09	4.45E-06
*TMEM50A*	1	Dup	58	36	0.21	0.08	3.19	1.94E-07	2.18E-04
*OR4F5*	1	Dup	22	6	0.08	0.01	6.65	6.31E-06	7.11E-03
*UGT2B15*	4	Dup	68	53	0.25	0.12	2.56	2.35E-06	2.65E-03
*ZNF595*	4	Dup	35	17	0.13	0.04	3.85	4.57E-06	5.15E-03
*ZNF718*	4	Dup	35	17	0.13	0.04	3.85	4.57E-06	5.15E-03
*OR2A1*	7	Dup	52	34	0.19	0.07	2.96	2.49E-06	2.80E-03
*OR2A42*	7	Dup	52	34	0.19	0.07	2.96	2.49E-06	2.80E-03
*CTAGE4*	7	Dup	53	36	0.20	0.08	2.85	3.85E-06	4.34E-03
*OR2A7*	7	Dup	53	36	0.20	0.08	2.85	3.85E-06	4.34E-03
*OR2A1*	7	Dup	52	36	0.19	0.08	2.79	6.80E-06	7.66E-03
*OR2A42*	7	Dup	52	36	0.19	0.08	2.79	6.80E-06	7.66E-03
*LONRF1*	8	Dup	10	0	0.04	0.00	Inf	4.49E-05	5.06E-02
*CBWD6*	9	Dup	17	3	0.06	0.01	10.14	1.17E-05	1.32E-02
*FOXD4L6*	9	Dup	17	3	0.06	0.01	10.14	1.17E-05	1.32E-02
*OR4K1*	14	Del	28	9	0.10	0.02	5.75	1.13E-06	1.27E-03
*LOC729355*	16	Dup	38	11	0.14	0.02	6.62	2.79E-09	3.14E-06
*TP53TG3*	16	Dup	38	11	0.14	0.02	6.62	2.79E-09	3.14E-06
*LOC729355*	16	Dup	40	20	0.15	0.04	3.79	1.23E-06	1.38E-03
*TP53TG3*	16	Dup	40	20	0.15	0.04	3.79	1.23E-06	1.38E-03
*LOC729355*	16	Dup	40	22	0.15	0.05	3.43	4.49E-06	5.06E-03
*TP53TG3*	16	Dup	40	22	0.15	0.05	3.43	4.49E-06	5.06E-03
*BAGE*	21	Dup	23	3	0.09	0.01	14.05	5.26E-08	5.92E-05
*BAGE2*	21	Dup	23	5	0.09	0.01	8.39	8.74E-07	9.84E-04
*BAGE3*	21	Dup	23	5	0.09	0.01	8.39	8.74E-07	9.84E-04
*BAGE4*	21	Dup	23	5	0.09	0.01	8.39	8.74E-07	9.84E-04
*BAGE5*	21	Dup	23	5	0.09	0.01	8.39	8.74E-07	9.84E-04

* One-sided Fisher's exact p value.

** P value Bonferroni-adjusted for 1126 genes overlapping CNVs called by both PennCNV and Birdsuite.

Gene-region is defined as gene ±20 kb.

Dup = duplication, Del = deletion.

### Rare CNVs

To test the hypothesis that cases have a higher burden of rare CNVs compared to controls, we examined rare CNVs called from both PennCNV and Birdsuite. We did not observe a significant excess of rare large CNVs or genes disrupted in BAVM cases compared to controls (data not shown).

PennCNV identified 542 genes with CNVs in BAVM cases but not in controls (CNV overlapping the gene ±20 kb). Birdsuite analysis identified 247 of these 542 genes. However, none of these genes were significantly associated with BAVM after correction for multiple testing. Table S3 lists thirteen BAVM-specific genes for which at least two BAVM subjects and no controls carried CNVs in both PennCNV and Birdsuite analysis. Eleven genes showed BAVM-specific deletions, while *ATG5* and *PRDM1* carried BAVM-specific duplications.

We also investigated whether BAVM cases carry a greater burden of rare CNVs overlapping genes in each of 7 candidate biological pathways relevant to BAVM based on prior studies (TGFβ signaling, Notch signaling, VEGF signaling, Angiogenesis, Vascular Development, Inflammatory Response and MAPK signaling). These pathways included a total of 572 genes. We did not observe statistically significant enrichment of rare CNVs in BAVM cases for any of the candidate pathways (data not shown).

### Gene ontology and functional annotation

The Database for Annotation, Visualization and Integrated Discovery (DAVID) v6.7b was used for analyzing functional classification, gene ontology of biological processes (GO), and pathway (http://david.abcc.ncifcrf.gov/) for the 542 genes bearing BAVM-specific CNVs using PennCNV. Several pathways, including the chemokine signaling pathway (15 genes, P = 1.2×10^−3^, Table S4) were nominally over-represented. In GO analysis, significantly enriched GO terms included positive regulation of smooth muscle cell proliferation (Fold enrichment = 9.89 and corrected P = 0.02) with a total of 8 AVM-specific genes (*PRKCA*, *TNF*, *NOTCH4*, *ITGA2*, *EGFR*, *AGER*, *FKBPL* and *AGPAT1*). The molecular function identified as most significant in GO analysis was cadmium ion binding (fold enrichment = 29.93, Bonferroni corrected P = 5.76×10^−7^, Table S5).

### Experimental validation

To validate the finding that BAVM cases have a higher burden of CNVs mapping to the 1p36.13 region encompassing the *NBPF1* gene compared to controls, we used several qPCR assays. First, we used a commercial qPCR assay targeting *NBPF1* (Applied Biosystems Taqman Hs04206910_cn). We observed a concordance rate of 78% between CNV states determined by qPCR and by PennCNV in 51 BAVM cases, including 23 called duplication and 28 called wildtype by PennCNV. For Birdsuite, we observed a concordance rate of 52% between Birdsuite calls and qPCR (14 called duplications and 37 called wildtype by Birdsuite). We then proceeded to replicate the 1p36.13 association with BAVM in an independent cohort of 184 BAVM cases and 182 healthy controls utilizing the qPCR assay. In the replication cohort, duplication at this locus was observed in 13% BAVM cases and 15% of the controls (OR = 0.81, P = 0.8) not supporting the original association results. Furthermore, we did not find support for replication utilizing 3 additional qPCR probes in the *NBPF1* gene region. Three of 177 BAVM cases were called duplications by all 3 probes, a much lower frequency than in the discovery cohort. The two replication cohorts contained 123 overlapping samples, and concordance between copy number calls was low (correlation r^2^ = 0.4).

## Discussion

This is the first genome-wide study to investigate whether CNVs might be associated with sporadic BAVM susceptibility. We identified several common CNV loci associated with sporadic BAVM in our Caucasian cohort of 371 sporadic BAVM cases and 563 controls. We focused on top findings that were in agreement between two CNV calling algorithms and between gene-based and segment-based analysis approaches. A BAVM-associated CNVR mapping to chr 1p36 was experimentally validated using quantitative real-time PCR, but did not replicate in an independent cohort of 184 BAVM cases and 182 controls.

The common BAVM-associated duplication observed at 1p36 encompasses *NBPF1*, the founding member of the *NBPF* gene family that consists of 22 genes and pseudogenes and likely arose by gene duplication. Very little is known about the function of *NBPF* proteins; some of them, including *NBPF1*, may be tumor suppressors [49]. Loss of heterozygosity (LOH) for the 1p36 locus encompassing the *NBPF1* gene has been shown in neuroblastoma and some other tumors [50]. To date, this region has not been reported to be associated with any vascular diseases or phenotypes.

Our initial association screen also identified other BAVM-associated CNVR mapping to chr15q11, 6q16 and 16p11. However, the association with BAVM did not persist in multivariate models adjusting for age, sex and the top 3 principal components, utilizing CNV calls from both PennCNV and Birdsuite. Further, none of these CNVRs overlapped genes associated with BAVM using the gene-based approach in both algorithms. The chromosome 15q11.2 CNVR identified in our study did not overlap the linkage region on 15q11-q13 reported in non-HHT familial BAVM patients [7]. The small deletion on 6q16.3 showed poor concordance between CNV-calling algorithms and did not overlap any genes. Due to the highly repetitive nature of the chr6 and 15 CNVR loci, we were not able to design qPCR probes for validation.

Although we did not observe a statistically significant association of rare CNVs with sporadic BAVM in our cohort, given our limited sample size, we cannot rule out the possibility that rare CNVs may contribute to BAVM susceptibility. All robust CNV associations to disease phenotypes that have been reported to date are with rare CNVs, with the exception of several autoimmune phenotypes [51], [52]. We identified 13 genes bearing BAVM-specific CNVs in at least 2 BAVM subjects, which are candidates for replication studies in larger BAVM cohorts. Notably, we did not identify any BAVM-specific CNVs in the 3 known HHT genes, in any other genes in the TGFβ signaling pathway, or in genes from 6 other biologically relevant pathways.

Functional classification, GO, and pathway analysis using genes exclusively deleted or duplicated in cases but not in controls identified several pathways and GO terms relevant to BAVM pathogenesis. The most significant pathway was the chemokine signaling pathway. Significantly enriched GO terms included positive regulation of smooth muscle cell proliferation and cadmium ion binding. Interestingly, studies of human vascular endothelial cells suggest cadmium may alter angiogenesis and induce apoptosis through VEGF signaling [53].

The size and the average number of CNVs called per individual differed significantly between cases and controls. In particular, when restricting to CNVs >10 kb in size, for deletions, the average number of CNVs per individual was significantly lower in cases than controls in both PennCNV and Birdsuite. This difference may be partially explained by differences in DNA extracted from blood and saliva. In our cohort, all control samples provided blood while some of the cases provided saliva specimens. It has been previously reported that CNV analyses differed between blood and saliva samples for the same individual, particularly for shorter CNV regions [54]. In contrast to a previous study [55], we identified a statistically significant increase in the number of CNVs detected in blood DNA specimens compared to saliva DNA specimens among cases. However, since case-control differences persisted even when restricting to blood DNA samples, the difference between saliva and blood DNA samples does not explain all of the observed effect. Furthermore, variation in experimental methods including time to genotype all study cases and controls (∼3 years) and batch (i.e., plate) effects may contribute to the observed case-control differences in the number of CNVs. While this is a limitation of our study, it would act as a negative confounder, since the excess of CNV calls was observed in controls, while the study hypotheses tested for an excess of CNVs in BAVM cases.

This study is limited by the small sample size, which was not powered to detect associations with small effect sizes. However, this is the largest cohort of sporadic BAVM patients for whom genome-wide genotype data have been analyzed. Further, we have only explored the role of CNVs in sporadic BAVM subjects of European ancestry; results may not generalize to other ethnicities. For the validation experiments, we evaluated the concordance of CNV states called between PennCNV, Birdsuite and qPCR among BAVM cases. Unfortunately, we were not able to evaluate the concordance in the controls used as we do not have access to their DNA. Since CNV calls from SNP arrays are based on relative signal intensity of a test sample compared to a reference, copy numbers in regions overlapping segmental duplications are highly variable and may not be reliably measured. In fact, copy number is unlikely to be 2 for a normal sample due to the repetitive nature of these regions. Our top finding is located in a region of segmental duplication and it is a known limitation that CNV calling algorithms may not reliably call CNVs overlapping segmental duplications, which comprise a large portion of the copy number variable regions in the human genome (∼29% [56]). This may explain why the association of the chr1p36 duplication with BAVM did not replicate in an independent cohort.

In conclusion, we provide the first evaluation of the role of CNVs in sporadic BAVM. We identified several candidate common CNV loci associated with BAVM, although the top finding on chromosome 1 did not replicate in an independent cohort. We also identified a number of genes bearing BAVM-specific CNVs; however, larger sample sizes are needed to test the hypothesis that rare CNVs contribute to BAVM pathogenesis.

## Supporting Information

Figure S1 **CNVs called by PennCNV that mapped to 1p36.13.** UCSC views of raw CNVs of type duplication called by PennCNV (BAVM cases in blue and controls in dark blue), mapping to the most significant BAVM-associated locus on 1p36.13 that encompasses the *NBPF1* gene. Depicted on the plot with a red arrow is the location of the Taqman Hs04206910_cn probe interrogating the *NBPF1* gene at Chr1:16802766 (NCBI build 36).
(TIF)

Table S1 **Characteristics of detected CNVs using PennCNV and Birdsuite (after QC).**
(DOCX)

Table S2 **Genes overlapping BAVM-associated CNVs using Birdsuite.**
(DOCX)

Table S3 **BAVM-specific genes with cases having at least two CNVs overlapping each gene identified by both PennCNV and Birdsuite.**
(DOCX)

Table S4 **Kegg pathways enriched among CNV-containing genes in BAVM cases.**
(DOCX)

Table S5 **Gene ontology categories enriched among CNV-containing genes in BAVM cases.**
(DOCX)
