# Supplementary figures and images for: A Genome-Wide Investigation of Copy Number Variation in Patients with Sporadic Brain Arteriovenous Malformation
Source: PLoS One. 2013 Oct 3;8(10):e71434. doi: 10.1371/journal.pone.0071434 (PMC3789669; doi:10.1371/journal.pone.0071434)

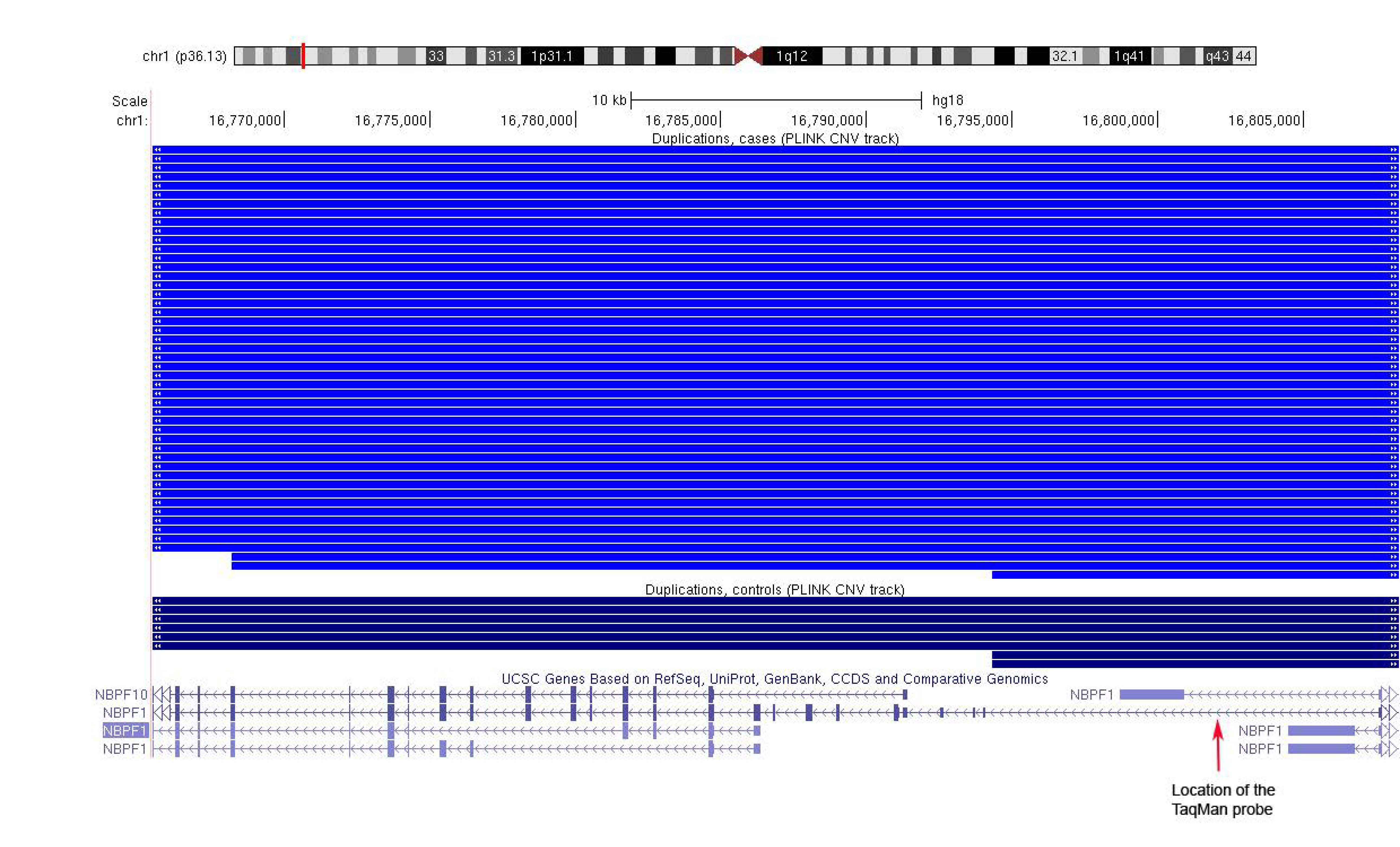

Supplement: Figure S1 — CNVs called by PennCNV that mapped to 1p36.13. UCSC views of raw CNVs of type duplication called by PennCNV (BAVM cases in blue and controls in dark blue), mapping to the most significant BAVM-associated locus on 1p36.13 that encompasses the NBPF1 gene. Depicted on the plot with a red arrow is the location of the Taqman Hs04206910_cn probe interrogating the NBPF1 gene at Chr1:16802766 (NCBI build 36). (TIF) [file pone.0071434.s001.tif]
