# Supplementary material for: A Genome-Wide Investigation of Copy Number Variation in Patients with Sporadic Brain Arteriovenous Malformation
Source: PLoS One. 2013 Oct 3;8(10):e71434. doi: 10.1371/journal.pone.0071434 (PMC3789669; doi:10.1371/journal.pone.0071434)
Supplement: Table S1 — Characteristics of detected CNVs using PennCNV and Birdsuite (after QC). (DOCX) [file pone.0071434.s002.docx]

**Table S1. Characteristics of detected CNVs using PennCNV and Birdsuite (after QC)**

| **PennCNV** | | | | |
| --- | --- | --- | --- | --- |
| **Type** | | **Cases (n=270)** | **Controls (n=457)** | **P*** |
| **Duplications** | |  |  |  |
| Total | | 3,934 | 6,928 |  |
| Mean number of CNV’s/subject (range) | | 14.57 (2-35) | 15.16 (4-42) | 0.22 |
| Median size in kb (range) | | 70,217 (1,030-26,490,472) | 47,700 (930-19,987,586) | < 2.2E-16 |
| **Heterozygous Deletions** | |  |  |  |
| Total | | 3,997 | 7,696 |  |
| Mean number of CNV’s/subject (range) | | 14.80 (4-58) | 16.84 (4-54) | 1.66E-11 |
| Median size in kb (range) | | 33,361 (1,021-24,009,077) | 32,681 (833-28,160,047) | 0.47 |
| **Homozygous Deletions** | |  |  |  |
| Total | | 1,307 | 2,493 |  |
| Mean number of CNV’s/subject (range) | | 4.91 (1-12) | 5.47 (1-13) | 0.001 |
| Median size in kb (range) | | 20,449 (1,030-332,486) | 17,339(1,199-389,583) | 0.122 |
| **Birdsuite** | | | | |
| **Type** | **Cases  (n=289)** | | **Controls  (n=443)** | **P*** |
| **Duplications** |  | |  |  |
| Total | 2,612 | | 3,735 |  |
| Mean number of CNV’s/subject (range) | 9.04 (2-19) | | 8.43 (2-27) | 0.001 |
| Median size in kb (range) | 108,838.5 (3,988-3,431,356) | | 97,339 (1,030-3,203,198) | 1.17E-04 |
| **Heterozygous Deletions** |  | |  |  |
| Total | 5,626 | | 10,479 |  |
| Mean number of CNV’s/subject (range) | 19.47 (5-39) | | 23.65 (3-43) | < 2.2E-16 |
| Median size in kb (range) | 27,075 (903 2,816,854) | | 25,330 (903-4,726,544) | 0.010 |
| **Homozygous Deletions** |  | |  |  |
| Total | 2,028 | | 3,177 |  |
| Mean number of CNV’s/subject (range) | 7.04 (1-14) | | 7.19 (2-14) | 0.422 |
| Median size in kb (range) | 20,628 (1,199-332,486) | | 21,109 (1,199-220,168) | 0.992 |

*P values are based on two tailed Mann-Whitney U test
