# Supplementary material for: A Genome-Wide Investigation of Copy Number Variation in Patients with Sporadic Brain Arteriovenous Malformation
Source: PLoS One. 2013 Oct 3;8(10):e71434. doi: 10.1371/journal.pone.0071434 (PMC3789669; doi:10.1371/journal.pone.0071434)
Supplement: Table S2 — Genes overlapping BAVM-associated CNVs using Birdsuite. (DOCX) [file pone.0071434.s003.docx]

**Table S2.**  **Genes overlapping BAVM-associated CNVs using Birdsuite**

| **Gene** | **Chr** | **Type** | **Cases** | **Controls** | **Proportion Cases** | **Proportion Controls** | **OR** | **P**  **(unadjusted)*** | **P (Bonferroni-adjusted) **** |
| --- | --- | --- | --- | --- | --- | --- | --- | --- | --- |
| *NBPF1* | 1 | Dup | 17 | 3 | 0.06 | 0.01 | 9.14 | 3.08E-05 | 3.47E-02 |
| *ZDHHC11* | 5 | Dup | 28 | 9 | 0.10 | 0.02 | 5.16 | 4.97E-06 | 5.60E-03 |
| *HLA-A29.1* | 6 | Dup | 99 | 80 | 0.34 | 0.18 | 2.36 | 5.98E-07 | 6.73E-04 |
| *HLA-A* | 6 | Dup | 99 | 80 | 0.34 | 0.18 | 2.36 | 5.98E-07 | 6.73E-04 |
| *LOC283755* | 15 | Dup | 54 | 32 | 0.19 | 0.07 | 2.95 | 2.88E-06 | 3.24E-03 |
| *NOMO1* | 16 | Dup | 90 | 75 | 0.31 | 0.17 | 2.22 | 6.21E-06 | 6.99E-03 |

*One-sided Fisher's exact p value ** P value Bonferroni-adjusted for 1126 genes overlapping CNVs called by both PennCNV and Birdsuite. Gene-region is defined as gene ± 20kb
