# Supplementary material for: A Genome-Wide Investigation of Copy Number Variation in Patients with Sporadic Brain Arteriovenous Malformation
Source: PLoS One. 2013 Oct 3;8(10):e71434. doi: 10.1371/journal.pone.0071434 (PMC3789669; doi:10.1371/journal.pone.0071434)
Supplement: Table S3 — BAVM-specific genes with cases having at least two CNVs overlapping each gene identified by both PennCNV and Birdsuite. (DOCX) [file pone.0071434.s004.docx]

**Table S3.**  **BAVM-specific genes with cases having at least two CNVs overlapping each gene identified by both PennCNV and Birdsuite**

|  |  |  |  | **PennCNV** | **Birdsuite** |
| --- | --- | --- | --- | --- | --- |
| **Gene** | **Chr** | **Start** | **End** | **cases** | **cases** |
| *ROPN1B* | 3 | 127,150,717 | 127,204,986 | 4 | 2 |
| *SLC41A3* | 3 | 127,187,889 | 127,305,824 | 4 | 2 |
| *ATG5* | 6 | 106,719,044 | 106,900,388 | 2 | 2 |
| *PRDM1* | 6 | 106,620,887 | 106,684,507 | 2 | 2 |
| *CNTNAP2* | 7 | 145,424,385 | 147,769,019 | 4 | 4 |
| *ASTN2* | 9 | 118,207,327 | 119,237,138 | 3 | 2 |
| *KRT33A* | 17 | 36,735,896 | 36,780,582 | 2 | 2 |
| *KRT33B* | 17 | 36,753,271 | 36,799,573 | 2 | 2 |
| *KRT34* | 17 | 36,767,446 | 36,812,162 | 2 | 2 |
| *CARD8* | 19 | 53,383,154 | 53,464,737 | 3 | 2 |
| *CYP2B6* | 19 | 46,169,043 | 46,236,141 | 2 | 2 |
| *C22orf25* | 22 | 18,368,630 | 18,453,447 | 2 | 2 |
| *DGCR8* | 22 | 18,427,833 | 18,499,400 | 2 | 2 |
