# Supplementary material for: A Genome-Wide Investigation of Copy Number Variation in Patients with Sporadic Brain Arteriovenous Malformation
Source: PLoS One. 2013 Oct 3;8(10):e71434. doi: 10.1371/journal.pone.0071434 (PMC3789669; doi:10.1371/journal.pone.0071434)
Supplement: Table S4 — Kegg pathways enriched among CNV-containing genes in BAVM cases. (DOCX) [file pone.0071434.s005.docx]

**Table S4.**  **Kegg pathways enriched among CNV-containing genes in BAVM cases**

| **Kegg pathway description** | **Count** | **%** | **Genes** | **Fold Enrichment** | **P** | **P (Bonferroni adjusted)** |
| --- | --- | --- | --- | --- | --- | --- |
| Chemokine signaling pathway | 15 | 0.34 | *CCL1, PARD3, CCL2, LYN, CCL8, ADRBK2, CCL7, CCL11, PTK2, CCL13, CCR6, ADCY9, GRK4, GNG4, AKT3* | 2.68 | 1.20E-03 | 0.16 |
| NOD-like receptor signaling pathway | 7 | 0.16 | *CCL11, CARD8, CCL13, TNF, CCL2, CCL8, CCL7* | 3.78 | 9.70E-03 | 0.75 |
| Hypertrophic cardiomyopathy (HCM) | 8 | 0.18 | *LAMA2, ACTC1, TNF, MYL3, ITGA8, PRKAB2, ITGA10, ITGA2* | 3.15 | 1.26E-02 | 0.84 |
| Cell adhesion molecules (CAMs) | 10 | 0.23 | *NCAM2, MPZL1, ITGA8, CD274, CNTNAP2, HLA-B, CDH4, HLA-DOB, HLA-DQA2, PDCD1* | 2.53 | 1.61E-02 | 0.90 |
| Dilated cardiomyopathy | 8 | 0.18 | *LAMA2, ACTC1, TNF, ADCY9, MYL3, ITGA8, ITGA10, ITGA2* | 2.91 | 1.89E-02 | 0.93 |
| Olfactory transduction | 19 | 0.43 | *OR2A2, OR7A17, OR2A12, OR51D1, OR2T2, OR2A14, OR1A1, OR1A2, OR2T3, ADRBK2, OR51E1, OR6C75, OR6C65, OR3A1, OR3A2, OR56B1, OR2T5, OR7C2, OR7A10* | 1.68 | 3.07E-02 | 0.99 |
| Type I diabetes mellitus | 5 | 0.11 | *TNF, HLA-B, HLA-DOB, HLA-DQA2, LTA* | 3.98 | 3.49E-02 | 0.99 |
| Asthma | 4 | 0.09 | *CCL11, TNF, HLA-DOB, HLA-DQA2* | 4.61 | 5.34E-02 | 1 |
| Allograft rejection | 4 | 0.09 | *TNF, HLA-B, HLA-DOB, HLA-DQA2* | 3.72 | 9.00E-02 | 1 |
| Antigen processing and presentation | 6 | 0.14 | *TAP2, TAP1, HLA-B, HLA-DOB, HLA-DQA2, LTA* | 2.42 | 9.94E-02 | 1 |
