# Supplementary material for: A Genome-Wide Investigation of Copy Number Variation in Patients with Sporadic Brain Arteriovenous Malformation
Source: PLoS One. 2013 Oct 3;8(10):e71434. doi: 10.1371/journal.pone.0071434 (PMC3789669; doi:10.1371/journal.pone.0071434)
Supplement: Table S5 — Gene ontology categories enriched among CNV-containing genes in BAVM cases. (DOCX) [file pone.0071434.s006.docx]

**Table S5. Gene ontology categories enriched among CNV-containing genes in BAVM cases**

| **Category** | **Term** | **Count** | **%** | **Genes** | **Fold Enrichment** | **P** | **P (Bonferroni-adjusted)** |
| --- | --- | --- | --- | --- | --- | --- | --- |
| GOTERM_BP_FAT | GO:0048661~positive regulation of smooth muscle cell proliferation | 8 | 0.2 | *PRKCA, EGFR, TNF, NOTCH4, ITGA2, AGER, FKBPL, AGPAT1* | 9.89 | 1.17E-05 | **0.02** |
| GOTERM_BP_FAT | GO:0051971~positive regulation of transmission of nerve impulse | 8 | 0.2 | *EGFR, LAMA2, TNF, CCL2, ITGA2, LTB, NFKBIL1, LTA* | 8.29 | 4.00E-05 | 0.08 |
| GOTERM_BP_FAT | GO:0046325~negative regulation of glucose import | 5 | 0.1 | *PRKCA, TNF, LTB, NFKBIL1, LTA* | 21.29 | 5.12E-05 | 0.10 |
| GOTERM_BP_FAT | GO:0031646~positive regulation of neurological system process | 8 | 0.2 | *EGFR, LAMA2, TNF, CCL2, ITGA2, LTB, NFKBIL1, LTA* | 7.86 | 5.71E-05 | 0.11 |
| GOTERM_BP_FAT | GO:0002037~negative regulation of L-glutamate transport | 4 | 0.1 | *TNF, LTB, NFKBIL1, LTA* | 38.32 | 6.85E-05 | 0.13 |
| GOTERM_BP_FAT | GO:0010829~negative regulation of glucose transport | 5 | 0.1 | *PRKCA, TNF, LTB, NFKBIL1, LTA* | 19.16 | 8.36E-05 | 0.15 |
| GOTERM_BP_FAT | GO:0002036~regulation of L-glutamate transport | 4 | 0.1 | *TNF, LTB, NFKBIL1, LTA* | 30.66 | 1.68E-04 | 0.29 |
| GOTERM_BP_FAT | GO:0048660~regulation of smooth muscle cell proliferation | 8 | 0.2 | *PRKCA, EGFR, TNF, NOTCH4, ITGA2, AGER, FKBPL, AGPAT1* | 6.66 | 1.70E-04 | 0.29 |
| GOTERM_BP_FAT | GO:0050806~positive regulation of synaptic transmission | 7 | 0.2 | *EGFR, LAMA2, TNF, CCL2, LTB, NFKBIL1, LTA* | 7.89 | 2.16E-04 | 0.35 |
| GOTERM_BP_FAT | GO:0044070~regulation of anion transport | 4 | 0.1 | *TNF, LTB, NFKBIL1, LTA* | 25.55 | 3.30E-04 | 0.48 |
| GOTERM_BP_FAT | GO:0051956~negative regulation of amino acid transport | 4 | 0.1 | *TNF, LTB, NFKBIL1, LTA* | 25.55 | 3.30E-04 | 0.48 |
| GOTERM_BP_FAT | GO:0009612~response to mechanical stimulus | 8 | 0.2 | *PRKCA, TXNIP, TNF, CCL2, ITGA2, LTB, NFKBIL1, LTA* | 5.47 | 5.89E-04 | 0.69 |
| GOTERM_BP_FAT | GO:0051953~negative regulation of amine transport | 4 | 0.1 | *TNF, LTB, NFKBIL1, LTA* | 19.16 | 8.88E-04 | 0.83 |
| GOTERM_BP_FAT | GO:0030030~cell projection organization | 21 | 0.5 | *PRKCA, EGFR, DCC, PARD3, LST1, TBCE, ALMS1, CDH4, CAPZB, SPAG16, EFHD1, NCAM2, PTK2, DNAI1, S100B, ULK1, ROBO1, ITGA8, TEKT4, LRRC50, RUNX3* | 2.19 | 1.57E-03 | 0.96 |
| GOTERM_BP_FAT | GO:0051247~positive regulation of protein metabolic process | 16 | 0.4 | *PRKCA, GDF2, TNF, LYN, BTRC, BMPR2, ITGA2, EGLN2, PSMB8, PSMB9, RNF180, PSMD13, PIAS3, AGT, TRIM32, DNMT3B* | 2.52 | 1.77E-03 | 0.97 |
| GOTERM_BP_FAT | GO:0032891~negative regulation of organic acid transport | 4 | 0.1 | *TNF, LTB, NFKBIL1, LTA* | 15.33 | 1.83E-03 | 0.97 |
| GOTERM_BP_FAT | GO:0051955~regulation of amino acid transport | 4 | 0.1 | *TNF, LTB, NFKBIL1, LTA* | 15.33 | 1.83E-03 | 0.97 |
| GOTERM_BP_FAT | GO:0019722~calcium-mediated signaling | 6 | 0.1 | *EGFR, TNF, ALMS1, LTB, NFKBIL1, LTA* | 6.21 | 2.55E-03 | 0.99 |
| GOTERM_BP_FAT | GO:0032268~regulation of cellular protein metabolic process | 24 | 0.5 | *PRKCA, EGFR, GDF2, TNF, LYN, BTRC, DDX1, BMPR2, ITGA2, ETF1, PRKCE, PSMB8, PSMB9, RNF180, PSMD13, ATG5, PIAS3, EIF3H, RBM8A, AGT, KRT7, EEFSEC, APOM, DNMT3B* | 1.94 | 3.18E-03 | 1.00 |
| GOTERM_BP_FAT | GO:0009628~response to abiotic stimulus | 20 | 0.5 | *PRKCA, EGFR, TXNIP, ARSB, RP1, TNF, CCL2, TRPM8, LYN, ITGA2, RCVRN, NFKBIL1, CCL11, TFEC, AGT, DNMT3B, LTB, LTA, FKBPL, OTOP1* | 2.08 | 3.59E-03 | 1.00 |
| GOTERM_BP_FAT | GO:0046677~response to antibiotic | 5 | 0.1 | *PRKCA, ACTR3, CYB5R4, CCL2, UQCRFS1* | 7.37 | 4.28E-03 | 1.00 |
| GOTERM_BP_FAT | GO:0032270~positive regulation of cellular protein metabolic process | 14 | 0.3 | *PRKCA, GDF2, TNF, LYN, BTRC, BMPR2, ITGA2, PSMB8, PSMB9, RNF180, PSMD13, PIAS3, AGT, DNMT3B* | 2.30 | 8.09E-03 | 1.00 |
| GOTERM_BP_FAT | GO:0032890~regulation of organic acid transport | 4 | 0.1 | *TNF, LTB, NFKBIL1, LTA* | 9.02 | 9.06E-03 | 1.00 |
| GOTERM_BP_FAT | GO:0045071~negative regulation of viral genome replication | 3 | 0.1 | *TNF, APOBEC3G, APOBEC3F* | 19.16 | 9.45E-03 | 1.00 |
| GOTERM_BP_FAT | GO:0031401~positive regulation of protein modification process | 12 | 0.3 | *PRKCA, GDF2, TNF, PSMD13, LYN, PIAS3, BTRC, AGT, BMPR2, DNMT3B, PSMB8, PSMB9* | 2.46 | 9.86E-03 | 1.00 |
| GOTERM_CC_FAT | GO:0016323~basolateral plasma membrane | 18 | 0.4 | *EGFR, NOX4, ENAH, HFE2, ARHGEF7, LPP, ITGA2, AGER, CTNNB1, ACTR3, PTK2, P2RY2, NOTCH4, CEACAM5, STK39, AFAP1, FKBPL, AGPAT1* | 3.23 | 4.32E-05 | **0.02** |
| GOTERM_CC_FAT | GO:0042825~TAP complex | 4 | 0.1 | *TAP2, TAP1, HLA-DOB, PSMB8* | 20.81 | 6.56E-04 | 0.21 |
| GOTERM_CC_FAT | GO:0042824~MHC class I peptide loading complex | 4 | 0.1 | *TAP2, TAP1, HLA-DOB, PSMB8* | 16.18 | 1.51E-03 | 0.42 |
| GOTERM_CC_FAT | GO:0005882~intermediate filament | 13 | 0.3 | *DLGAP2, GAN, KRT34, KRT33A, KRT33B, KRT80, KRT7, KRTAP19-5, KRTAP19-6, KRTAP19-3, KRTAP19-4, KRTAP19-1, KRTAP19-2* | 2.59 | 4.56E-03 | 0.80 |
| GOTERM_CC_FAT | GO:0009925~basal plasma membrane | 5 | 0.1 | *ACTR3, NOTCH4, AGER, FKBPL, AGPAT1* | 7.00 | 5.13E-03 | 0.84 |
| GOTERM_CC_FAT | GO:0045111~intermediate filament cytoskeleton | 13 | 0.3 | *DLGAP2, GAN, KRT34, KRT33A, KRT33B, KRT80, KRT7, KRTAP19-5, KRTAP19-6, KRTAP19-3, KRTAP19-4, KRTAP19-1, KRTAP19-2* | 2.53 | 5.41E-03 | 0.85 |
| GOTERM_CC_FAT | GO:0045178~basal part of cell | 5 | 0.1 | *ACTR3, NOTCH4, AGER, FKBPL, AGPAT1* | 6.07 | 8.63E-03 | 0.95 |
| GOTERM_CC_FAT | GO:0042995~cell projection | 31 | 0.7 | *DCC, PARD3, ENAH, AIF1, GNA12, CAPZB, SPAG16, CTNNB1, ACTR3, PTK2, DNAI1, ROBO1, ROPN1B, EXOC4, KLHL24, TEKT4, LRRC50, DLGAP2, ARHGEF7, ALMS1, ITGA2, GAPDHS, NCAM2, S100B, ULK1, PIAS3, ITGA8, DLD, GRK4, MAPRE1, FBXO10* | 1.62 | 9.52E-03 | 0.97 |
| GOTERM_MF_FAT | GO:0046870~cadmium ion binding | 8 | 0.2 | *MT1M, MT1A, MT1E, MT1B, MT1H, MT1G, MT1X, MT1F* | 29.93 | 1.01E-09 | **5.76E-07** |
| GOTERM_MF_FAT | GO:0050785~advanced glycation end-product receptor activity | 4 | 0.1 | *NOTCH4, AGER, FKBPL, AGPAT1* | 37.41 | 7.36E-05 | **0.04** |
| GOTERM_MF_FAT | GO:0005507~copper ion binding | 9 | 0.2 | *TYR, MT1M, MT1A, MT1E, MT1B, MT1H, MT1G, MT1X, MT1F* | 4.88 | 4.76E-04 | 0.24 |
| GOTERM_MF_FAT | GO:0008009~chemokine activity | 6 | 0.1 | *CCL1, CCL11, CCL13, CCL2, CCL8, CCL7* | 4.88 | 7.31E-03 | 0.98 |
| GOTERM_MF_FAT | GO:0051287~NAD or NADH binding | 6 | 0.1 | *GPD1L, GAPDHS, DLD, IDH3B, GRHPR, SIRT3* | 4.78 | 8.01E-03 | 0.99 |
| GOTERM_MF_FAT | GO:0042379~chemokine receptor binding | 6 | 0.1 | *CCL1, CCL11, CCL13, CCL2, CCL8, CCL7* | 4.58 | 9.53E-03 | 1.00 |
